# Abnormal diastolic and systolic septal motion following pericardiectomy demonstrated by ciné DENSE MRI

**Published:** 2008

**Authors:** BRUCE SPOTTISWOODE, ERNESTA M MEINTJES, JAMES B RUSSELL, BONGANI M MAYOSI, SULAIMAN MOOSA, FREDERICK H EPSTEIN

**Affiliations:** MRC/UCT Medical Imaging Research Unit, University of Cape Town, Observatory; MRC/UCT Medical Imaging Research Unit, University of Cape Town, Observatory; Department of Medicine, Groote Schuur Hospital and University of Cape Town, Observatory; Department of Medicine, Groote Schuur Hospital and University of Cape Town, Observatory; Department of Radiology, Groote Schuur Hospital and University of Cape Town, Observatory; Departments of Radiology and Biomedical Engineering, University of Virginia, USA

## Abstract

Constrictive pericarditis can lead to paradoxical interventricular septal motion. Displacement encoding with stimulated echoes (DENSE) magnetic resonance imaging (MRI) provides a method for quantifying myocardial motion and strain. A case of constrictive pericarditis is presented and the diastolic ‘septal bounce’ is clearly evident in both anatomical and DENSE ciné MRI images. (See video link to full-text electronic article). The postoperative systolic septal wall-motion abnormality of cardiac surgery is portrayed with greater precision by DENSE than anatomical ciné MRI images.

## Summary

Constrictive pericarditis is present when a fibrotic, thickened, and adherent pericardium restricts diastolic filling of the heart. In early diastole, when intracardiac volume is less than that defined by the stiff pericardium, diastolic filling is unimpeded, and early diastolic filling occurs abnormally rapidly because venous pressure is elevated. The rapid early diastolic filling which is halted abruptly when intracardiac volume reaches the limit set by the non-compliant pericardium is reflected by the abrupt displacement of the interventricular septum into the left ventricle (LV) during early diastole (ie, the septal bounce).^1^ By contrast, abnormal septal systolic motion is a well-known phenomenon following cardiac sugery.^2^

## Case report

A 47-year-old man presented on 6 January 2006 with signs and symptoms of pericardial effusion. Pericardiocentesis yielded 550 ml of straw-coloured fluid, which grew *Mycobacterium tuberculosis* after 16 days of culture, thus confirming a diagnosis of tuberculous pericarditis. He was commenced on standard anti-tubercular medication for six months and a tapering course of adjunctive oral prednisone for six weeks. He did not respond as expected to this treatment, such that on 3 March 2006 he developed clinical and echocardiographic features of effusive constrictive pericarditis. Pericardiectomy was performed on 20 July 2006. Post-operative examination in September 2006, however, demonstrated clinical features of persistent constrictive pericarditis and no bundle branch block on the electrocardiogram.

**Fig. 1.**
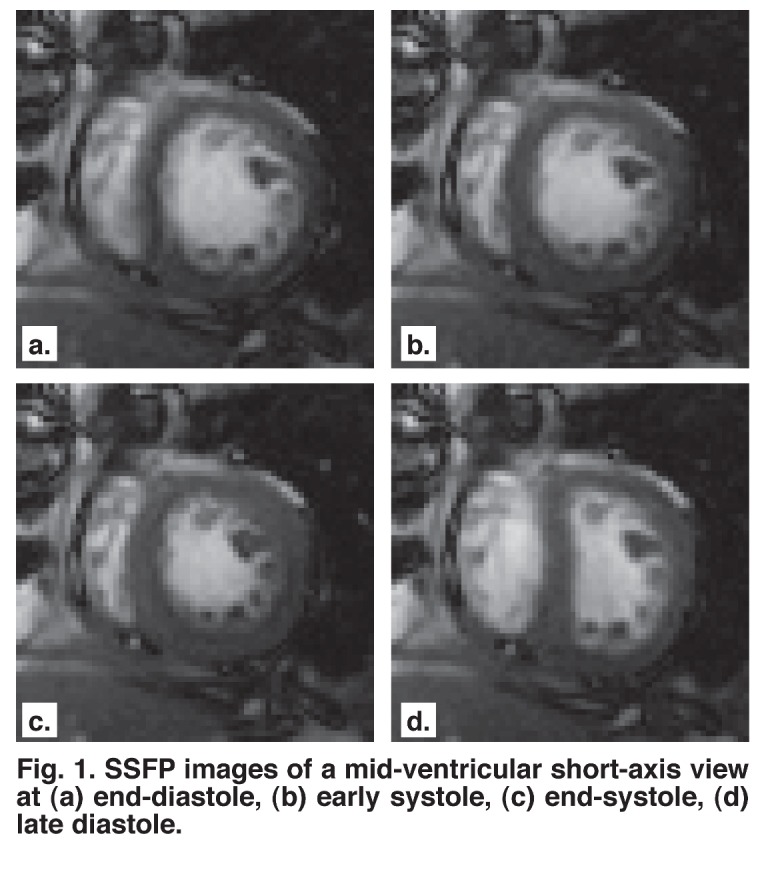
SSFP images of a mid-ventricular short-axis view at (a) end-diastole, (b) early systole, (c) end-systole, (d) late diastole.

Magnetic resonance imaging (MRI) was performed on 5 October 2006, showing the diastolic ‘septal bounce’ of constriction on steady-state free precession (SSFP) imaging in a midventricular short-axis view [Fig. 1, Movie 1 (see link to full-text electronic article)]. Displacement encoding with stimulated echoes (DENSE) was incorporated to quantify the ventricular septal motion in diastole and systole.

DENSE^3^ is an MRI technique that non-invasively measures intramyocardial displacement at a high spatial resolution, and ciné DENSE^4^ provides a time series of these measurements. Discrete portions of myocardium can be tracked through time and myocardial deformation or strain can be derived from the resultingmotion trajectories.^5^ Ciné DENSE images of a mid-ventricular short-axis view were acquired using the same parameters as described previously.^6^ Delayed-enhancement phase-sensitive inversion recovery (PSIR) images were also acquired at the same slice but no unusual myocardial enhancement was noted.

**Fig. 2.**
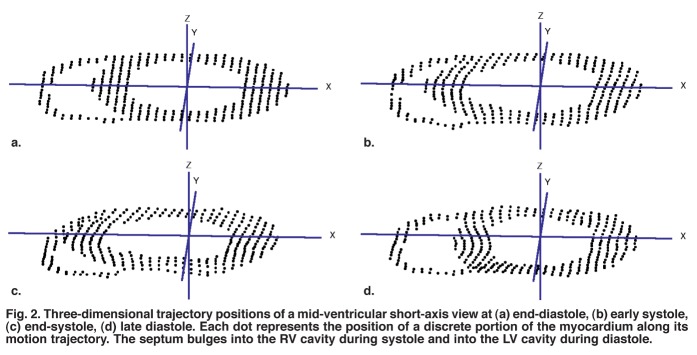
Three-dimensional trajectory positions of a mid-ventricular short-axis view at (a) end-diastole, (b) early systole, (c) end-systole, (d) late diastole. Each dot represents the position of a discrete portion of the myocardium along its motion trajectory. The septum bulges into the RV cavity during systole and into the LV cavity during diastole.

The ciné DENSE motion trajectory positions at four cardiac phases from a mid-ventricular short-axis slice are shown in Fig. 2; Movie 2 shows these trajectory positions for all cardiac phases. The septum is seen to bulge paradoxically into the RV cavity during systole and then into the LV cavity during diastole. The septal bulging into the RV cavity during systole is not clearly shown in the SSFP images (Fig. 1, Movie 1). A measure of strain orientated tangentially to the mid-wall was calculated for both the LV and RV, as described previously.^6^

**Fig. 3.**
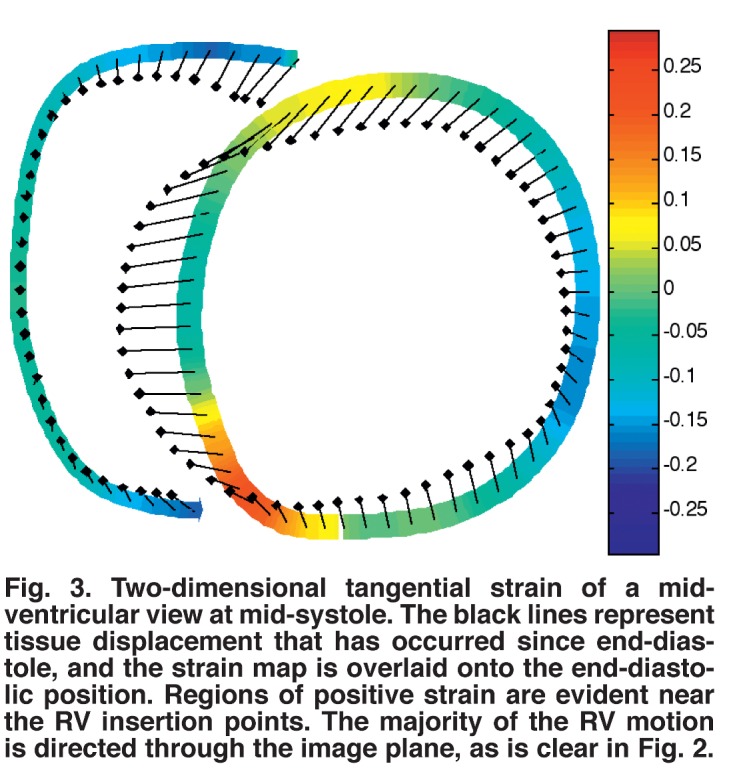
Two-dimensional tangential strain of a midventricular view at mid-systole. The black lines represent tissue displacement that has occurred since end-diastole, and the strain map is overlaid onto the end-diastolic position. Regions of positive strain are evident near the RV insertion points. The majority of the RV motion is directed through the image plane, as is clear in Fig. 2.

Fig. 3 shows the tangential strain (which corresponds to circumferential shortening in the LV) for this patient at midsystole; Movie 3 shows tangential strain throughout the cardiac cycle. The septum is shown bulging into the RV cavity and regions of positive strain are evident near the RV insertion points. It seems reasonable to anticipate high stresses (and consequently, strains) at the hinge points of this unruly septal motion. No unusual strain patterns were noted during diastole.

The patient was treated conservatively with anti-tubercular medication and diuretics, and his signs and symptoms gradually improved without further surgical intervention. Ciné DENSE is shown to define the dominant postoperative systolic septal wall motion abnormality of cardiac surgery with greater precision than anatomical ciné MRI images.
